# Vaccination and COVID-19 in Polish Dialysis Patients: Results from the European Clinical Dialysis Database

**DOI:** 10.3390/vaccines10091565

**Published:** 2022-09-19

**Authors:** Wojciech Marcinkowski, Konrad Zuzda, Jacek Zawierucha, Tomasz Prystacki, Paweł Żebrowski, Jacek S. Małyszko, Ewa Wojtaszek, Jolanta Małyszko

**Affiliations:** 1 Fresenius Nephrocare, Krzywa 13, 60-118 Poznan, Poland; 2 Department of Nephrology, Dialysis and Internal Diseases, Medical University of Warsaw, 02-097 Warsaw, Poland; 3 1st Department of Nephrology and Transplantology, Medical University of Bialystok, 15-540 Bialystok, Poland

**Keywords:** dialysis, mortality, COVID-19

## Abstract

**Background:** Patients with end-stage kidney disease undergoing hemodialysis are particularly vulnerable to severe COVID-19 as a result of older age and multimorbidities. **Objectives:** Data are still limited and there are no published data on mortality in hemodialyzed patients in Poland, in particular when vaccines became available. We assessed the epidemiologic and clinical data of patients with laboratory-confirmed COVID-19 and assessed the mortality in 2019, 2020, and 2021, as well as the vaccination rate in 2021. **Patients and Methods:** Retrospectively collected data from 73 Fresenius Nephrocare Poland hemodialysis centers and one public unit were analyzed. **Results:** In 2021, the vaccination rate was 96%. The unadjusted mortality (number of deaths divided by number of patients) in 2019 was 18.8%, while the unadjusted (after exclusion of COVID-related deaths) mortality in 2020 was 20.8%, and mortality in 2021 was 16.22%. The prevalence of cardiovascular deaths in 2019 and 2020 was almost identical (41.4% vs. 41.2%, respectively), and in 2021, the figures increased slightly to 44.1%. The prevalence of sudden cardiac deaths in 2019 was higher than in 2020 (19.6% vs. 17.3%, respectively) and consequently decreased in 2021 (10.0%), as well as strokes (6.2% vs. 5.4%, and 3.31% in 2021), whereas deaths due to gastrointestinal tract diseases were lower (2.5% vs. 3.2%, and 2.25% in 2021), diabetes complications (0.5% vs. 1.3%, and 0.5% in 2021), sepsis (5.1% vs. 6.3%, and 8.79% in 2021), respiratory failure (1.2 vs. 1.6%, and 2.83% in 2021), and pneumonia (1.4% vs. 2.0%, and 0.82%). There were 1493 hemodialyzed COVID-19 positive patients, and among them, 191 died in 2020 (12.79%). In 2021, there were 1224 COVID-19 positive patients and 260 died (21.24%). The mortality of COVID-19 positive dialyzed patients contributed 13.39% in 2020 and 16.21% in 2021 of all recorded deaths. **Conclusions:** The mortality among HD patients was higher in 2021 than in 2020 and 2019, despite the very high vaccination rate of up to 96%. The higher non-COVID-19 mortality may be due to the limited possibility of hospitalization and dedicated care during the pandemic. This information is extremely important in order to develop methods to protect this highly vulnerable patient group. Prevention plays a key role; other measures are essential in the mitigation and spread of COVID-19 in HD centers.

## 1. Background

Severe acute respiratory syndrome coronavirus 2 (SARS-CoV-2) spread worldwide at an alarming rate since the outbreak began in China in December 2019 [1]. A total of 79.2 million people were infected with SARS-CoV-2 by the end of 2020 and 1.7 million died [2]. According to the information provided by the Ministry of Health in Poland, the number of reported coronavirus disease 2019 (COVID-19) cases by 9 September 2022 was 6,213,262, and there were 117,252 deaths (https://www.gov.pl/web/koronawirus/wykaz-zarazen-koronawirusem-sars-cov-2, accessed on 12 September 2022). COVID-19 infection poses a unique challenge in dialysis patients, especially for those treated at an HD facility. This disease interferes mainly with the respiratory system and potentially causes death. Older age, coexisting diabetes, hypertension, organ failure, and coagulation disruption are risk factors for adult respiratory distress syndrome and death [3]. The clinical presentation of the infection can differ from the more frequent asymptomatic condition to a mild and non-specific respiratory distress syndrome [4].

Patients with uremia are vulnerable to infection and may demonstrate greater infectivity and variability in clinical manifestations [5]. Hemodialysis (HD) dramatically increases the risk of transmission, including among medical staff and facility employees, the patients themselves, and their families [6]. Additionally, the pandemic instilled concerns in patients’ minds about visiting dialysis centers. COVID-19 is suspected to cause significant mortality in the dialysis population due to chronic kidney disease and the prevalence of comorbidities such as diabetes and cardiovascular disease. Precocious measures, i.e., hygiene and isolation, social distancing, and lockdowns were not sufficient to prevent the spread of COVID-19, and there was a need for the urgent introduction of vaccinations, in particular to vulnerable populations and healthcare professionals, in order to halt the pandemic as soon as possible.

## 2. Objective

This study aimed to explore the impact of the COVID-19 pandemic on hemodialysis patients before and after the vaccines became available. We compared multiple characteristics in HD populations from 14 March 2020, when the first case of COVID-19 in Poland was registered, until 31 December 2021. We also explored the vaccination rate in our dialysis population.

## 3. Patients and Methods

### 3.1. Patients

The reported data came from 66 of 73 centers of Fresenius Medical Care (FMC) Poland from the European Clinical Database (EuCliD) system. Fresenius Medical Care Poland is the largest private dialysis provider, operating 73 HD and PD centers countrywide. At the end of 2021 in Poland, 18,479 patients were on HD in 180 non-public units and 98 public units. In addition, 890 patients were on peritoneal dialysis in 31 non-public units and 49 public units. The healthcare services provided by FMC are financed by the National Health Fund (Polish Public Payer) and are available for all patients suffering end-stage CKD. In FMC Poland, about 30% of all HD patients (*n* = 5564 in 2021) and about 22% of all PD patients (*n* = 198 in 2021) are dialyzed; therefore, the data are representative for our country population.

### 3.2. Methods

Demographic (age and sex) and clinical data (concomitant diseases, time from the diagnosis of kidney disease, time from the onset of renal replacement therapy to death, and time from the start of RRT in the unit in months) were collected throughout 2019, 2020, and 2021. Each dialysis center was asked to report all new cases of COVID-19 and all deaths due to the disease since the beginning of the pandemic until 31December 2021. All cases of SARS-CoV-2 infection were confirmed by nasopharyngeal/oropharyngeal swabbing with the reverse transcriptase–polymerase chain reaction test (PCR) method. We did not use antigen tests to confirm infection. In addition, antibodies in either class IgG or class IgM were not tested. The virus genotype (variant) was not checked. We also collected data on the comorbidities and causes of death using ICD-codes. The assessment of whether or not death was due to COVID-19 was performed by the healthcare personnel at each dialysis center. In principle, testing in the majority of units was prompted by suggestive clinical signs. Single dialysis units periodically tested their entire dialysis population. It is almost impossible to reliable clarify the role of SARS-CoV-2 infection as the cause of death in dialyzed patients due to the additional factors existing in the analyzed population (comorbidities and age). For the purposes of this study, the death of patients infected SARS-CoV-2 were treated as death caused by COVID-19. We also collected the types of vaccines used in the dialysis patients.

### 3.3. Statistical Analysis

Descriptive analysis was performed to highlight the differences between groups. Continuous variables were presented as mean and compared with the t-test. The Chi-square test was used to compare the categorical variables, where applicable. A two-tailed *p* value of less than 0.05 was considered significant. The general adult population of Poland was selected as a reference. Statistical analysis was performed using GraphPad Prism version 9.3.0 for macOS (GraphPad Software, San Diego, CA, USA). The institutional review board at the Medical University of Warsaw, Poland, does not require informed consent for retrospective studies based on medical records. The study was in compliance with the Helsinki Declaration (https://www.wma.net/what-we-do/medical-ethics/declaration-of-helsinki (accessed on 1 August 2022)).

## 4. Results

**Figure 1:**
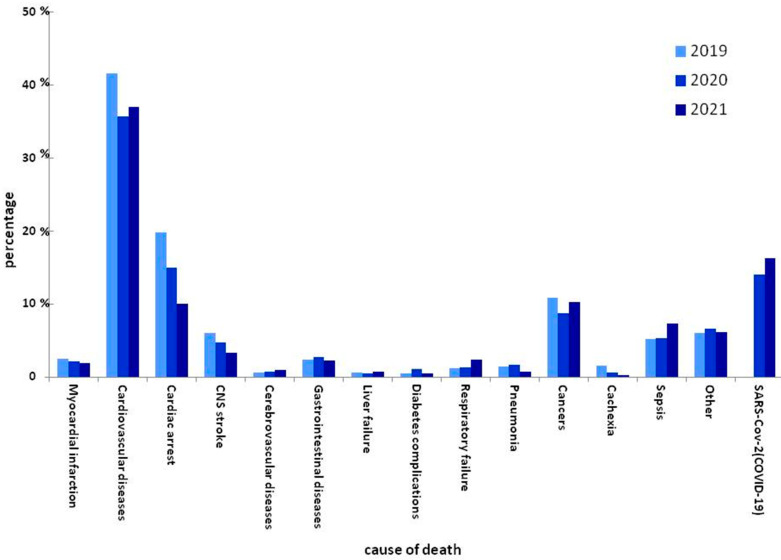
Comparison of causes of death between 2019, 2020, and 2021.

Over 90.41% of dialysis units provided their data. The average annual number of patients between 2019 (5901) and 2020 (5886) differed slightly (Δ15). The total number of HD patients exposed chronically to COVID-19 in the studied period was 5886. The number of deaths for 2019 was 1089 (18.5%), and for 2020 it was 1426 (23.8%). Of these, in 2020, 191 (115 men, 76 females) patients died from COVID 19. Excluding COVID-19, in 2020, the number of deceased patients was 1235 (20.3%). The mortality rate in the hemodialyzed patients in 2019, 2020, and 2021 in comparison with the general adult population of Poland from 14 March 2020 until 31 December 2021 are provided in Table 1. The crude mortality rate due to COVID-19 per 1000 HD patients was 31.90 compared with 0.88 per 1000 adult patients from the general adult population in 2020 (*p* < 0.001). In 2021, the crude mortality rate due to COVID-19 per 1000 HD patients increased to 47.28 compared with 2.16 per 1000 adults from the general population (*p* < 0.001). Comparing the crude mortality rate without COVID-19 deaths between the general population in 2020, HD population in 2019, and HD population in 2020, the following values were observed: 13.96, 184.54, and 206.32, respectively. Comparing the mortality between 2019 and 2020 (including death due to COVID-19), it was significantly different (*p* < 0.001). However, during the comparison of mortality without deaths due to COVID-19, there was still a significant increase in mortality in 2020 (*p* < 0.001). In 2021, the figures were significantly higher. When comparing the crude mortality rate without COVID-19 deaths between the general adult population, HD population in 2019, HD population in 2020, and HD population in 2021, the authors observed the following values: 14.44, 184.54, 206.32, and 244.22, respectively. Even excluding COVID-19 deaths in 2021, when vaccinations were available, the crude mortality was higher than in previous years, and the same applied in the general population. Figure 1 shows the causes of death in dialyzed patients over the three consecutive years. In 2020, COVID-19 was responsible for 13.4% (*n* = 191) of deaths in the HD population. In Table 2, the proportion of specific cause categories in the total number of deaths is presented. Analyzing other causes of death, including the ratio of fatalities without COVID in 2020 to the ratio of deaths in 2019, the ratio increased in cerebrovascular diseases (1.5x), gastrointestinal diseases (1.3x), complications of diabetes (2.6x), respiratory failure (1.2x), pneumonia (1.4x), sepsis (1.2x), and other (1.3x).

In the second analyzed period (2021), COVID-19 caused 16.21% (*n* = 260) deaths in the hemodialyzed population. For other causes of death without SARS-CoV-2 infections, the ratio increased in liver and respiratory failure (1.8x and 1.9x, respectively).

The basal data in mortality in 2019, 2020, and 2021 in relation to COVID-19 are given in Table 3. All-cause mortality, including COVID-19, in 2021 and 2020 was higher than in 2019 (28.96% and 23.8% vs. 18.5%, respectively, *p* < 0.01), and mortality without COVID-19 was also higher in 2020 and 2021 vs. 2019 (20.6% and 24.26% vs. 18.5%, respectively, *p* < 0.05). Mortality in relation to age is given in Table 4. The mean patient age at the time of death in 2019 was 72.59, in 2020 it was 72.01, and in the COVID-19 group it was 72.67. In 2021, the mean patients age at time of death was 71.4 and 72.26, respectively. Time from renal replacement therapy initiation to death, measured in months, in 2019 was 58, in 2020 it was 61, in 2021 it was 50 months, and in the COVID-19 group it was 62 in 2020 and 49 months in 2021. The sex percentages among the deceased were as follows: in 2019, 38.4% were women and 61.6% were men; in 2020, 40.7% and 59.3%; in 2021, it was 40.0% and 60%; and in the COVID-19 group it was 39.8% and 60.2%, respectively. A similar distribution was observed in 2021, at 39.61% and 60.39%, respectively. Data are given in Table 5.

We also analyzed the vaccination rate in 2021 when vaccines were available for dialyzed patients and kidney transplant recipients as a priority, together with patients from nursing homes and health care professionals. In 2021, the vaccination rate was 96%, with the majority being vaccinated with Comirnaty (Pfizer-BioNTech https://www.ema.europa.eu/en/medicines/human/EPAR/comirnaty (accessed on 1 August 2022)) (*n* = 5440), followed by Moderna, The National Institute of Allergy and Infectious Diseases (NIAID), USA (https://www.ema.europa.eu/en/documents/product-information/spikevax-previously-covid-19-vaccine-moderna-epar-product-information_pl.pdf (accessed on 1 August 2022)) (*n* = 136); Astra Zeneca, Oxford, UK (https://www.ema.europa.eu/en/medicines/human/EPAR/vaxzevria-previously-covid-19-vaccine-astrazeneca (accessed on 1 August 2022) (*n* = 50); and Janssen, Belgium (https://www.ema.europa.eu/en/medicines/human/EPAR/jcovden-previously-covid-19-vaccine-janssen (accessed on 1 August 2022)) (*n* = 26). We did not check seropositivity in our patients.

## 5. Discussion

In our study, the idea was to show that in our dialysis population, the vaccination rate was 96%, as we could not find any data about such a high rate in the published literature. In general, published data considered a small group and the response rate was measured by the titers of the antibodies. As FMC covered about 30% of all HD patients in Poland and 22% of all PD patients, these data reflect the situation in our country and are representative. According to the data from https://www.gov.pl/web/szczepimysie/raport-szczepien-przeciwko-covid-19 accessed on 13 September 2022 of the Ministry of Health website, full vaccination was reported in 51.41% of the entire Polish population. We would like to stress the enormous success rate of vaccination in the dialysis population in Poland in relation to the general population. This was probably also the most significant observation in the dialyzed population, including PD patients, published to date, covering the entire period since the beginning of the pandemic throughout two waves of SARS-CoV-2 until the commencement of population vaccinations, which took place in our region at the end of December 2020. Nevertheless, the results obtained require careful interpretation, bearing in mind the following limitations. In addition, in our university hospital, the vaccination rate in peritoneal dialysis patients was 100% and only one patient died before the vaccine was introduced. In HD patients, the vaccination rate was 96%, which is in contrast with the recent report from Egypt, where more than half (out of 237) of HD patients received the COVID-19 vaccine. Vaccine acceptability is not associated with age, gender, or educational level, but rather with employment status and residency [9]. However, the prevalence of vaccinations was not provided. We also would like to stress that in recent studies on the efficacy of the vaccination in HD, number of patients enrolled were small [10,11,12,13]. In a national multicenter observational cohort performed in Chile on a total of 12,301 HD patients, 10,615 (86.3%) received complete vaccination (two doses) [14]. In our population, 96% received three doses. 

The SARS-CoV-2 pandemic forced changes in the functioning of many medical entities around the world. From the beginning of the pandemic, most dialysis providers implemented several solutions to maximize the protection of HD patients against the viral exposition. The main solutions were infected patient isolation (temporal and spatial), changing the HD schedule and HD session duration (transient shortening the session time), additional disinfection procedures (including disinfection of ambulances), and separation during transportation. Patients, medical staff, and service staff (e.g., drivers and assistants) were obliged to wear the mask during transportation (at least FFP2), admission, and the entire dialysis session. The body temperature was measured in all patients prior to entering the dialysis center. The specific HD treatment (4h of HD in the center three times a week, and a much longer stay in the HD unit while waiting for transport or for the start of HD) limits possible protection measures, and it was thus impossible to limit the personal contact between patients and medical staff or to separate every patient.

The early termination of restrictive measures increases the risk of another wave [15], and any new disease-related data would be beneficial to improve care. Since the onset of the COVID-19 pandemic, there has been debate regarding whether HD patients are at a higher risk of fatality from COVID-19. Hemodialysis patients are a particular group of patients exposed to the severe course of SARS-CoV-2 infection due to immune deficiency [5] in general, irrespective of it being the leading cause of ESRD. To aid efforts in the management of the COVID-19 pandemic, it is essential to understand the epidemiology of the disease.

Very early findings from Wuhan implied a milder presentation of the disease in these patients [16]. The results of the European Renal Association–European Dialysis and Transplant Association Registry database study, developed to prospectively collect specific records of individual European dialysis patients with COVID-19, demonstrated that COVID-19-related case mortality rate after 28 days of follow-up was 20.0% in 3285 patients receiving dialysis [17]. Data from the annual report of the Polish Nephrology Registry [18] mentioned in the study by Puchalska-Reglińska et al. [19] suggested a significant increase in overall mortality among 1567 HD patients from 14 units of the Pomeranian Voivodeship in 2020, reaching 25%, and COVID-19 was responsible for over 30% of all deaths. In our study, these values were 23.5% and 13.4%, respectively, in a significantly larger group of patients (*p* < 0.001), and were similarly described in other reports from Spain, Scotland, and Brazil [20,21,22]. In comparison, the overall mortality in the HD population reported in this study in 2019 was 18.5%.

The mortality rate in our cohort was superior to that in the general COVID-19 population—31.91 compared with a mortality rate of 0.88 in the general COVID-19 population. This disorder appeared to have a more significant impact on the HD population relative to the general population [7,8]. The male gender was found to influence mortality in HD patients, which was also seen in the general Polish population. The correlation in patients on long-term hemodialysis was also noted in several studies [17]. This supports previous findings in the general population, as well as the slightly increased cardiovascular mortality found in older men versus women on dialysis without COVID-19 [23,24]. Hence, the absence of such a correlation in long-term HD patients was reported in studies from Ontario and the UK, as well as other smaller cohorts in Poland [19,25,26].

Of course, the comparison of a relatively small, very specific population such as the dialyzed population, with a general one has some limitations (different age structure, health status, and even gender distribution). The intention of the authors was to show that SARS-CoV-2 infection poses a significantly greater threat to life among dialyzed patients in comparison with the general population. Despite an almost 100% vaccination rate, mortality in 2021 in dialyzed patients remained high. However, from clinical experience, we did not note severe COVID-19 infection or ICU admissions.

The main strength of the study is the fact that we included data from over 30% of the dialyzed Polish population. As they came from single chain FMC and our public unit, they were representative. Patients were given treatment using standard protocols and standard precaution measures. In addition, our analysis covered the entire period of the pandemic in Poland caused by the classic virus version. The SARS-CoV-2 Alpha variant appeared in February 2021 and became dominant in March 2021 in Poland. It had a higher transmission rate and would probably significantly alter epidemiological indicators. We also included data on the vaccination rate and type of vaccine in 2021 in the population studied as a novelty.

### Limitations

The overall applicability of this research is restricted by the limited number of patients included, and the fact that the findings may have been influenced by elevated stress levels caused by constant media coverage of the spread and dangers of the coronavirus [27]. There were 278 dialysis units in Poland in 2021 (98 were public and 180 were non-public, including 73 owned by FMC), of which we were able to obtain data from 66 of them, representing 23.74% of the total, treating 30.1% of HD patients. Our study did not take into account the number of infected patients and the resulting incidence rate. The genotype of COVID-19 was not tested. As the first COVID-19 case in Poland was registered in March 2020, the observation period in 2020 was 2 months shorter than whole year of 2019 (used for comparison). Taking this fact into consideration can predict that the mortality among dialyzed patients in 2020 was significantly higher than that reported in this study. The full year of data for 2021 clearly showed that mortality among HD patients significantly increased during the COVID-19 pandemic, despite the excellent vaccination rate, in practically all of the HD patients studied. This could also be due to a weaker cellular immune response, which was not measurable using serum.

## 6. Conclusions

Patients receiving maintenance hemodialysis are susceptible to COVID-19, and hemodialysis centers were high-risk settings during the pandemic. However, changes in HD schemes due to the necessity to isolate COVID-19 positive patients through shortened dialyses did not change the cardiovascular mortality significantly in the hemodialyzed population. All-cause mortality including COVID-19 was higher in 2020 and 2021, despite the extremely high vaccination rate in 2021, reaching 96%. The higher death rate due to sepsis, pneumonia, and respiratory failure could be attributed, to some extent, to COVID-19 infection, whereas mortality due to the complications of diabetes and gastrointestinal disorders may be due to limited access to primary care physicians, hospitalization, and dedicated care during the pandemic. Telemedicine, however helpful [28], resulted in delayed care on the one hand, and patients were also afraid to go to outpatient clinics or hospital due to fear of COVID-19. The overloaded healthcare system caused significant delays in complication treatment, which was one of the additional factors of increased mortality. Despite the high vaccination rate, reaching 96%, the mortality in 2021 was still high. This may be due to the lower seropositivity after vaccination in the HD population, as reported in some studies [29,30,31]; however, the vaccination rates in these studies were not provided and the sample sizes were small. However, the validity of the test for the assessment of the levels of antibodies against COVID-19 is still a matter of debate. Even though vaccination did not prevent COVID-19 infection, it instead resulted in a less severe course, requiring hospital admission less often and very seldom ICU admission. Similar findings were reported recently by Esposito et al. [32] in a small population of HD patients.

Therefore, we will struggle with the legacy of pandemic in the next couple of years. COVID-19 became a huge challenge and danger for the dialyzed population. This is extremely important to consider in order to develop methods to protect this highly vulnerable patient group. Prevention plays a key role, but other measures are essential in the mitigation and containment of the COVID-19 pandemic in HD centers. Time and resources are needed to lower the mortality in dialyzed patients in the post-pandemic era and to provide better access to health care system for patients with chronic kidney disease in order to slow the progression of the disease, in particular not to reach end-stage kidney disease, and the need for renal replacement therapy. This is of the utmost importance, as the number of kidney transplantations also declined during the pandemic. Beyond regular protective procedures such as patients and medical staff using personal protective equipment, frequent and careful disinfection of all surfaces, and isolation of infected patients, vaccinations are strongly recommended for immunocompromised patients, including those on renal replacement therapy.

## Figures and Tables

**Table 1:** Comparison of the mortality rate in hemodialyzed patients in 2019, 2020, and 2021 and in the adult general population of Poland from 14 March 2020 until 31 December 2021.

	Hemodialysis Population in 2019	Hemodialysis Population in 2020	Hemodialysis Population in 2021	Adult General Population 2020 [7,8]	Adult General Population 2021
Overall adult population, n	5901	5886	5534	32,139,021	31,223,400
COVID-19 cases, n	N/A ^B^	1493	1224	1,294,878	2,813,449
COVID-19 deaths, n	N/A ^B^	191	260	28,554	67,698
Overall deaths, n	1089	1426	1603	477,355	518,664
Deaths without COVID, n	1089	1235	1343	448,801	450,966
Mean age of deceased patients, y (SD)	72.59 (12.38)	72.01 (12.34)	71.41 (12.41)	76.65 (N/D) ^A^	72.61
COVID-19 mortality rate per 1000, crude	N/A ^B^	31.91	47.28	0.88	2.16
Mortality rate per 1000 excluding COVID-19, crude	184.54	206.32	244.22	13.96	14.44

^A^—Not done. ^B^—Not applicable.

**Table 2:** The proportion of specific cause categories in the total number of deaths.

Cause	Hemodialysis Population in 2019	%	Hemodialysis Population in 2020	%	Hemodialysis Population in 2021	%	Hemodialysis Population in 2020 (%) without COVID Deaths	Hemodialysis Population in 2021 (%) without COVID Deaths	The Proportion of Deaths without COVID in 2020 to the Proportion of Deaths in 2019	The Proportion of Deaths without COVID in 2021 to the Proportion of Deaths in 2020
Myocardial infraction	27	2.48%	30	2.10%	30	1.87%	2.43%	2.23%	1.0	1.2
Cardiovascular Diseases	453	41.60%	508	35.62%	593	36.99%	41.13%	44.15%	1.0	1.1
Cardiac arrest	215	19.74%	214	15.01%	161	10.04%	17.33%	11.99%	0.9	0.7
CNS Stroke	66	6.06%	67	4.70%	53	3.31%	5.43%	3.95%	0.9	0.7
Cerebrovascular diseases	6	0.55%	10	0.70%	16	1.00%	0.81%	1.19%	1.5	1.5
Gastrointestinal diseases	26	2.39%	39	2.73%	36	2.25%	3.16%	2.68%	1.3	0.8
Liver failure	7	0.64%	6	0.42%	12	0.75%	0.49%	0.89%	0.8	1.8
Diabetes complications	5	0.46%	15	1.05%	8	0.50%	1.21%	0.60%	2.6	0.5
Respiratory failure	13	1.19%	18	1.26%	38	2.37%	1.46%	2.83%	1.2	1.9
Pneumonia	15	1.38%	24	1.68%	11	0.69%	1.94%	0.82%	1.4	0.4
Cancers	118	10.84%	125	8.77%	164	10.23%	10.12%	12.21%	0.9	1.2
Cachexia	17	1.56%	9	0.63%	4	0.25%	0.73%	0.30%	0.5	0.4
Sepsis	56	5.14%	76	5.33%	118	7.36%	6.15%	8.79%	1.2	1.4
Other	65	5.97%	94	6.59%	99	6.18%	7.61%	7.37%	1.3	1.0
SARS-CoV-2 (COVID-19)	N/A	N/A	191	13.39%	260	16.22%	N/A	N/A	1.0	1.0
Total	1089	100%	1426	100%	1603	100%	100%	100%	N/A	N/A

**Table 3:** Basal data in mortality in 2019, 2020, and 2021 in relation to COVID-19.

Basal Data	Year		
	2019	2020	2021
Deaths (n)	1089	1426	1603
Annual numer of patients	5901	5886	5534
Mortality (%)	18.5	23.8 **	28.96 ***
COVID-19 deaths (n)	0	191	260
Mortality without COVID-19 (%)	18.5	20.6 *	24.26 **

* *p* < 0.05, ** *p* < 0.01, *** *p* < 0.001.

**Table 4:** Basal data on the total mortality and COVID-19 mortality in 2019, 2020, and 2021 in relation to age.

2019	Mortality Total	COVID	2020	Mortality Total	COVID	2021	Mortality Total	COVID
Age <45	25	0	<45	52	3	<45	53	2
Age 45–64	214	0	45–64	268	37	45–64	363	32
Age ≥65	850	0	≥65	1106	151	≥65	1188	154

**Table 5:** Characteristics of deceased patients in 2019, 2020, and 2021 in relation to COVID-19.

	2019	2020	2021	2020 without Patients Died of COVID-19	2021 without Patients Died of COVID-19	2020 Died of COVID-19	2021 Died of COVID-19
Patients, n	1089	1426	1603	1235	1343	191	260
Mean age, y (SD)	72.59 (12.38)	72.01 (12.34)	71.4 (12.42)	71.91 (13)	72.26 (12.46)	72.67 (11)	72.26 (11.76)
Time from the onset of RRT to death (months)	58	61	50	61	49	62	56
Time from the start of RRT in the unit (months)	48	51	50	51	49	54	56
Time from diagnosis of kidney disease (months)	81	94	71	92	71	101	69
Female, n	418	580	642	504	532	76	110
Female (%)	38.4	40.7	40.0	40.8	39.6	39.8	42.3
Male, n	671	846	961	731	811	115	150
Male (%)	61.6	59.3	60.0	59.2	60.4	60.2	57.7

## Data Availability

Not applicable.
